# Do dog breeds differ in pain sensitivity? Veterinarians and the public believe they do

**DOI:** 10.1371/journal.pone.0230315

**Published:** 2020-03-17

**Authors:** Margaret E. Gruen, Philip White, Brian Hare

**Affiliations:** 1 Department of Evolutionary Anthropology, Duke University, Durham, North Carolina, United States of America; 2 Department of Statistical Science, Duke University, Durham, North Carolina, United States of America; 3 Center for Cognitive Neuroscience, Duke Institute for Brain Sciences, Duke University, Durham, North Carolina, United States of America; Universidade do Porto Instituto de Biologia Molecular e Celular, PORTUGAL

## Abstract

Humans do not respond to the pain of all humans equally; physical appearance and associated group identity affect how people respond to the pain of others. Here we ask if a similar differential response occurs when humans evaluate different individuals of another species. Beliefs about pain in pet dogs (*Canis familiaris*) provide a powerful test, since dogs vary so much in size, shape, and color, and are often associated with behavioral stereotypes. Using an on-line survey, we asked both the general public and veterinarians to rate pain sensitivity in 28 different dog breeds, identified only by their pictures. We found that both the general public and veterinarians rated smaller dogs (i.e. based on height and weight) as being more sensitive to pain; the general public respondents rated breeds associated with breed specific legislation as having lower pain sensitivity. While there is currently no known physiological basis for such breed-level differences, over 90% of respondents from both groups indicated belief in differences in pain sensitivity among dog breeds. We discuss how these results inform theories of human social discrimination and suggest that the perception of breed-level differences in pain sensitivity may affect the recognition and management of painful conditions in dogs.

## Introduction

Humans do not respond to the suffering of all other humans the same way [1]. One person seeing another in pain will not always perceive that the pain experience would be equal for different people in identical situations (e.g. [2]). This can lead to differential treatment of individuals and groups, with far reaching consequences. Humans typically use differences in physical appearance, ethnicity, socioeconomic status, or behavioral stereotypes to justify explicit beliefs that some groups or individuals do not experience pain equally. These differences have been attributed to a host of psychological causes including prejudice, conformity, and even dehumanization [3].

The healthcare system in the United States is not immune to these effects. Disparities exist in the US with regard to the treatment and management of pain across individuals [4, 5]. The root causes of the disparity in pain management may be linked to such issues as access to healthcare [6, 7]; however, other research suggests that people’s perception of pain sensitivity in others may be affected by biases that may in turn influence pain recognition and management. The best studied are the effects of gender [8–10] and race [4, 11–17] on ratings of pain, with research suggesting that physician assessment of pain level of an individual can be influenced by the race of the patient [4, 11, 12]. Importantly, this difference exists without explicit racial bias on the part of the physician [13, 15]. Indeed, racially based disparities in ratings of pain sensitivity are not restricted to physicians, but have also been shown in ratings by general population members [14, 17, 18].

The presence of this disparity in pain sensitivity ratings shows that perception of pain in another person can be influenced by phenotypic variation, and that medical training does not necessarily eliminate this bias [15]. The ability to perceive and ascribe emotions, including pain, to another being, human or non-human, is a feature of empathy [19]. Empathy, generally, is influenced by how the target entity is perceived, and by features of both the empathizer and the target. These features include group membership and similarity to self, likeability, and trustworthiness [20, 21]. Humans can also feel empathy, including assessment of pain, toward non-human species [22], and personal empathy has been associated with higher ratings of pain in non-human species including cattle [23] and dogs [24].

While these studies have evaluated human ratings of pain for several non-human species, none have evaluated ratings of pain sensitivity *within* a non-human species. Dogs represent an important species for evaluating perceptions of pain sensitivity. Due both to their status as social companions and their relevance as models of pain in humans [25–27], studying dogs allows us to explore the extension of empathy to interspecific social partners. Dogs, as a species, are rated as high in both warmth and competence [28]. However, among dogs there exists a broad diversity of phenotypic variation that affects how dogs are perceived in terms of temperament and behavior. Breed biases and stereotypes are pervasive [29] with breed status affecting adoption [30, 31] and breed-specific legislation directed at particular breeds.

Belief in breed differences is high; purebred status remains an important consideration in dog ownership [32] and scores of books have been published on breed characteristics. While breed differences have been noted in susceptibility to disease [33], including diseases identified as painful [25], no literature exists investigating breed differences in pain sensitivity, or in the estimation of human beliefs regarding such differences. Individual variability in pain sensitivity certainly exists [34], but this is not known to be a breed-wide phenomenon (i.e. within breed variability is greater than between breed variability). Theoretically, all dogs should have similar pain-related neurobiology, regardless of breed.

We put forward the Generalized Group Characterization (GGC) hypothesis which posits, despite evidence for physiological similarity across dog breeds, that the human perception of pain sensitivity in dogs is heavily influenced by group psychology. Just as these empathic responses are heavily influenced by implicit or explicit characterizations of human groups, they are similarly influenced when responding to animal pain [28, 35]. This leads to the prediction that salient features that distinguish breed groups, like body size, will be related to ratings of pain sensitivity. We further predict that other social traits typically attributed to specific groups of dogs will influence pain ratings. For example, dog breeds typically stereotyped as being more threatening/potentially harmful or less trustworthy (i.e. those singled out by breed-specific legislation [BSL]) will be rated as having lower pain sensitivities than less threatening dog breeds. These breeds vary by local jurisdiction; for this survey we included: Pitbull, Doberman Pinscher, English Mastiff, and Rottweiller. The term Pitbull (with various spellings) is a grouping of dogs of a number of registered breeds (American Pit Bull Terrier, American Staffordshire Terrier and Staffordshire Bull Terrier, among others) but is also applied to any dog with a particular phenotype (large square head, muscular but medium size, short coat). For this survey, ‘Pitbull’ was represented by an American Staffordshire Terrier. The null hypothesis predicts that people perceive pain sensitivity similarly across dog breeds.

To provide a test of the GGC hypothesis, we developed a survey that asked respondents to rate the pain sensitivity of different dog breeds while looking at a representative picture from that breed. We varied the size and shape of breeds we presented and included several breeds typically viewed as more threatening. To create the opportunity to examine what experiential factors might help explain any differential rating of pain in dogs we observe, we surveyed members of the general public and veterinarians. While we made no *a priori* predictions regarding how these two groups might rate pain in different dogs, we predicted both groups would provide differential ratings. However, given one group has much more experience dealing with dogs in pain, we tested how training and experience as a veterinarian might shape the perception of pain in dogs by people.

## Methods

The survey instrument was composed using standardized survey software (Qualtrics®), and two forms of the survey (A and B) were constructed. Twenty-eight dog breeds were selected to encompass a range of sizes, body and head shapes, and coat types/lengths [36]. Pictures of dogs from each of these breeds were selected and standardized so that they all faced the same direction. A scale was added by the investigator to provide a reference for height, and was set according to the breed standard for height at the withers (www.akc.org/dog-breeds/).

Forms A and B were identical for 22 breeds; for six breeds, two color variants were shown (S1 Fig in S1 File). For the survey, respondents were asked to, “Please rate the following breeds along a scale that ranges from ‘Not at all sensitive’ to ‘Most sensitive imaginable.’ Please answer based on what you have observed or would predict.” Below each picture, an 11-point rating scale was provided that ranged from Not at all sensitive = 0 to Most sensitive imaginable = 100; respondents indicated their choice by selecting a radio button. The veterinary version of the survey was constructed following the same format as the general public version, including the same pictures of the dogs and an identical rating scale. Due to differences in software, the veterinary version of the survey differed in several small ways. First, only one coat color was shown for each of the dog breeds; the pictures selected matched the general public form B. Second, as the distribution network was different, some of the demographics questions presented after the dog breed ratings were not necessary; a question about year of graduation from veterinary school was added. Otherwise, the survey instruments were identical, and respondents were allowed to skip questions if they desired. Further details of the dogs and survey preparation are described in supplementary materials and the survey (form B) is shown in S2 Fig in S1 File.

For both the general public and veterinary versions, informed consent was gathered prior to the start of the survey. The surveys were presented in seven blocks as follows: 1) informed consent; 2) an explanation of the rating scale and example; 3) presentation of the 28 dog breeds and rating scale, with breeds presented in random order; 4) questions regarding respondents’ beliefs in differences in pain sensitivity between dog breeds and reasons for these differences; 5) demographic questions; 6) a feelings thermometer, where respondents were asked to rate how warmly or coolly they felt about different groups of dogs on a scale of 0 (cool) to100 (warm) with 50 as neutral; and 7) an opportunity to provide feedback about the survey in general. For the dog breed ratings, respondents were able to skip any questions, though they were reminded that they had not filled them in when they advanced to the next section. Forced responses were used for the demographic questions (i.e. respondents could not advance without completing these questions). Demographics collected from the general public respondents included: gender, race, age, education level, and experience with dogs. Veterinarian respondents were asked to provide gender and graduation year. The protocol and survey were reviewed by the Duke University Institutional Review Board and granted exemption (E0245). All participants were required to be over 18 years of age to be eligible. Both studies were conducted on populations within the United States of America; all methods were performed in accordance with the relevant guidelines and regulations.

### Survey populations

#### General public

The survey was distributed to members of the general public (non-veterinarians) using a professional survey service (Survey Sampling International; Shelton, CT) in order to obtain a representative sample of 1000 complete responses (500 each for form A and B).

#### Veterinarians

The survey was distributed to veterinarians through the Veterinary Information Network. This membership-based service is the largest national and international network of veterinarians. Surveys were emailed to members, and participation was voluntary.

### Statistical analysis

Raw data were exported from the survey software in tabular form. Data were evaluated to ensure a single response for each respondent (using ID number or IP address). Incomplete surveys were removed if respondents skipped >50% (14) dog breeds. Feedback columns were assessed, and individual responses removed when a clear misunderstanding had occurred. Free choice text responses were reviewed and grouped by keyword. Descriptive statistics were used for respondent demographics, with the following modifications to the independent variables:

- Gender was treated as binary, excluding respondents who did not indicate male or female.
- Race (general public only) was categorized as Caucasian, African-American, Hispanic, and other.
- Graduation year (Veterinarians only) was categorized as 2015–2016; 2010–2014; 2000–2009, 1990–1999; 1980–1989; <1980.

Respondent age and feelings thermometer ratings were entered as continuous variables, pain sensitivity ratings for self (general public only) and for each dog breed were entered as ordinal level variables, and education and dog experience were entered as categorical variables. Dog breeds were evaluated individually, and also by their presence or absence on breed-specific legislation (see S1 Table in S1 File). The average height and weight for each dog breed (from the breed standard) were included and analyzed individually and as an interaction between height and weight. Finally, level of agreement with belief statements and attribution of contribution of specific factors on dog breeds’ pain sensitivity and response were considered ordinal variables.

Due to the ordinal nature of the pain sensitivity ratings, proportional-odds cumulative logit models were fit and models compared using ANOVA to analyze the need for subject specific random intercepts, form (A vs B) effects, dog breed effects, and the difference in variance and pain ratings between the general public and the veterinarian respondents. To account for multiple comparisons between these models, a Bonferroni correction was used and significance was set at α<0.0062. Two-sample t-tests were used to compare the ratings of the general public and veterinarians for individual dog breeds. Separate cumulative logit models were fit for the general public and the veterinarians to simultaneously evaluate the effects of available human demographic features (gender, age, education, race, experience with dogs, and graduation year category) and dog features (height, weight, height*weight, BSL). For these regression models, α<0.05 was considered significant. The relationship between the strength of belief that dog breeds differ in their sensitivity to pain and a respondent’s standard deviation across dog breeds was evaluated using Bonferroni corrected pairwise t-tests. For this analysis only, we evaluated pain sensitivity ratings as a continuous variable [15, 18] in order to calculate a standard deviation for each respondent and display a mean-based ranking of breeds for the general public and veterinarians. Finally, separate cumulative logit models were fit for the general public and the veterinarians to evaluate the relationship between the feelings thermometer ratings and pain sensitivity ratings. Models were fit for each of the four breeds where feelings thermometer corresponded to a single breed-specific pain sensitivity rating (Golden retriever, Greyhound, Pitbull, and Pug). The remaining feelings thermometers were for categories of dogs; dog breeds were grouped by size (small/toy, medium/large, and giant) and models fit for each of these categories. For these models, a Bonferroni correction was used and significance was set at α<0.0071. All analyses were carried out using the ordinal package in R (R Core Team [2013]).

#### Data availability

The datasets and computer code generated during and/or analyzed during the current study are available from: https://doi.org/10.5061/dryad.r2280gb9j.

## Results

We report results from the general public survey, the veterinarian survey, and a comparison between the general public and veterinarian surveys.

### General public survey

A total of 1053 survey responses were included in the analyses, 521 from form A and 532 from form B. Demographic characteristics were similar for respondents of forms A and B for gender, age, education level, race, and experience with dogs (See supplementary materials [S2 Table in S1 File] for further details).

#### Pain sensitivity ratings for dog breeds

Pain sensitivity ratings were provided for all dog breeds by the vast majority of respondents, with no more than two missing values for any of the 28 breeds. The general public respondents rated sensitivity in dog breeds along a spectrum from not at all sensitive to most sensitive imaginable.

Compared to a model including BSL status, dog features, and human demographics, inclusion of subject as a random effect (LR = 13419; df = 1; p < 2.2e^-16^), breed as a random effect (LR = 661; df = 3; p < 2.2e^-16^), form (A vs. B; p < 2.2e^-16^), and a breed*form interaction (LR = 1210; df = 1; p < 2.2e^-^16) each significantly improved model fit.

Forms A and B displayed two color variants for Chihuahuas, Pomeranians, Great Danes, Schnauzers, Dachshunds, and Chow Chows. The difference in log-odds between form A and form B for these six breeds are shown in supplementary materials (S3 Table in S1 File). Results suggest that the differences between the forms are driven by Chihuahuas and Pomeranians, with Great Danes very close to significantly different between the two forms. In all three of these breeds, form A showed a dark color variant of the breed, which was rated as less sensitive to pain than the light color variant shown in form B.

#### Pain sensitivity related to participant and dog variables

Results for participant variables are described in supplementary materials. Significant effects were found for BSL (z = -3.20, p < 0.001) with dogs on the BSL list being rated as having lower pain sensitivity than those not on the BSL list, scaled dog weight (z = -3.49, p = 0.0005), and a scaled dog weight*height interaction (z = 5.50, p < 0.0001), such that heavier dogs were rated as less sensitive to pain, but dogs who were both lighter and smaller and dogs who were both heavier and taller were rated as more sensitive to pain. No significant effects were found for scaled dog height.

### Veterinarian survey

A total of 1078 survey responses were included in the analyses. Distribution of gender and graduation year category are shown in Table 1. Further details on the respondents are described in supplementary materials.

**Table 1:** Distribution of the gender and graduation year categories for respondents to the veterinary survey.

n = 1,100	Gender		Graduation year (by category)						
	Female	Male	2015–2016	2010–2014	2000–2009	1990–1999	1980–1989	<1980	Missing
Total	842	236	121	188	230	217	220	86	16
Percentage	78.1%	21.9%	11.2%	17.4%	21.3%	20.1%	20.4%	8.0%	1.5%

#### Pain sensitivity ratings for dog breeds

Pain sensitivity ratings were provided by at least 90% of respondents for all 28 breeds, with at least 95% in 21 of 28 breeds (75%).

#### Pain sensitivity related to participant and dog variables

Relative to graduation in 2015–2016, all other graduation categories had negative z values, indicating lower pain sensitivity ratings, though the only the category to reach significance was for graduation years 1980–1989 where respondents rated dogs as less sensitive overall (z = -2.13, p = 0.03). A significant effect was found for scaled dog height*weight interaction (z = 2.11, p = 0.03), such that both lighter/shorter and heavier/taller dogs were rated as more sensitive to pain. No significant effects were found for BSL (z = -1.79, p = 0.07), gender, or scaled dog height or weight.

### Comparisons between the general public and veterinary surveys

**Fig 1:**
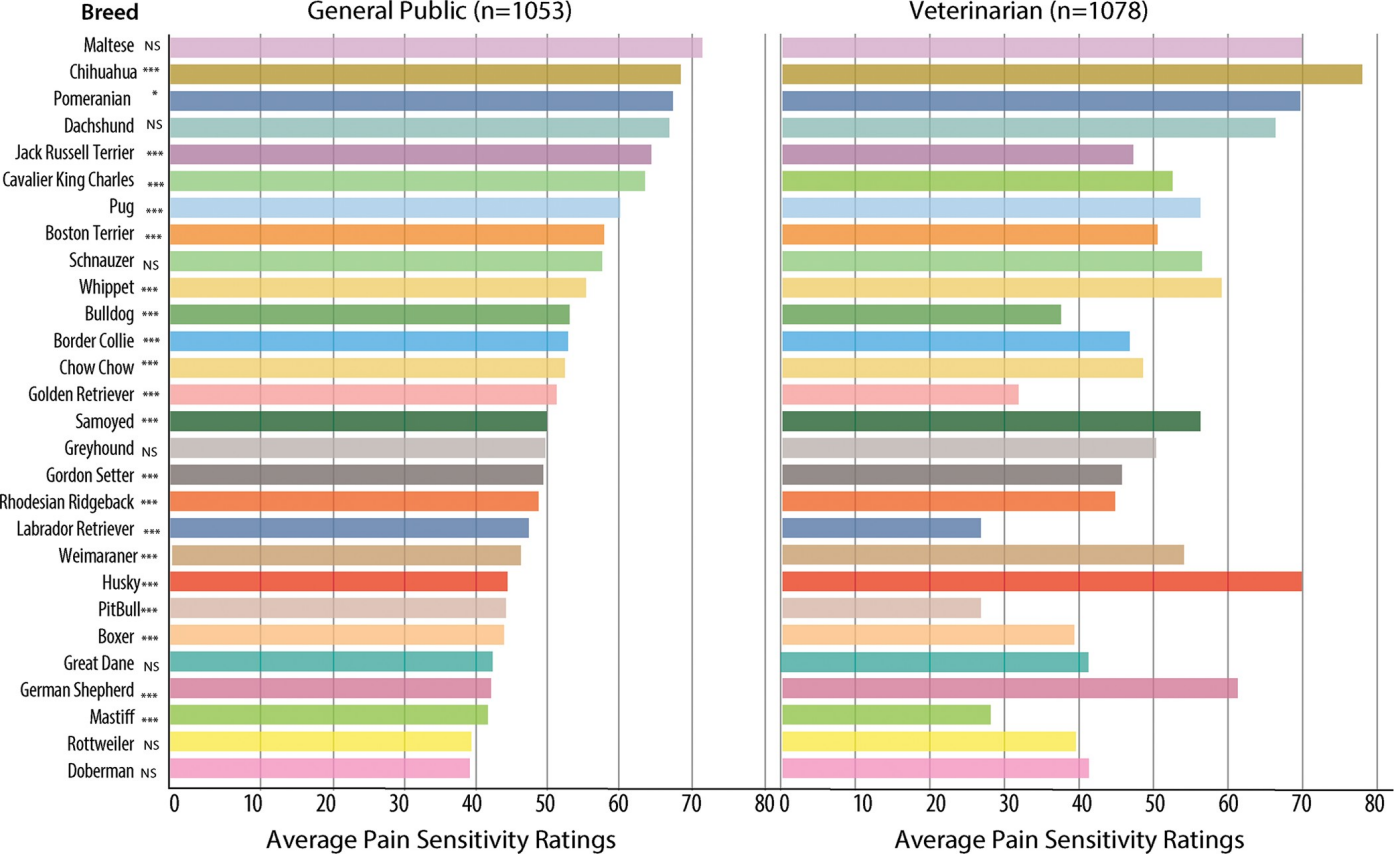
Average pain sensitivity ratings for each dog breed, shown separately for the general public and veterinarian respondents. The scale ranged from 0 = not at all sensitive to 100 = most sensitive imaginable. Median pain sensitivity ratings between the veterinarians and general public were compared using two-sample t-tests. p-values are *** = 0.001, * = 0.05.

Median ratings for pain sensitivity were significantly different between the general public and veterinarians for the majority of breeds (Fig 1). Marked differences were found for particular breeds, where the median ratings were on opposite sides of the middle of the scale (50). These breeds included the German Shepherd dog and the Husky (both rated as significantly less sensitive to pain by the general public respondents than the veterinarian respondents). Comparison between the ratings of the general public and veterinarian responses was highly significant (LR = 330.31; df = 6; p < 2.2e^-16^) with veterinarians rating dog breeds as less sensitive overall than the ratings from the general public. In addition, the standard deviation of the general public respondents’ random intercepts was 1.47, compared with 0.55 for the veterinary respondents, showing that the veterinarians’ ratings were more consistent with each other than were the general public respondents’ ratings.

#### Beliefs regarding differences in pain sensitivity and reaction to pain

Overall, agreement that dog breeds differ in their sensitivity to pain was high among both the general public (94.0%) and the veterinarians (98.3%). Similarly, agreement that dog breeds differ in their response to pain was high among the general public (95%) and veterinarians (100%).

All respondents who indicated agreement that dog breeds differ in sensitivity or response to pain rated the relative contribution of several factors to a breed’s sensitivity and response to pain. Not all respondents rated extent for all factors; thus, results are shown as the number and percentage of those who responded on that factor in Table 2. Categorization of free-text responses for the factor “Other” and results for response to pain are described in supplementary materials (S4 Table in S1 File). Veterinarians and general public members attributed similar influence of genetics on pain sensitivity, but general public respondents attributed greater influence to developmental environment and skin thickness on pain sensitivity and less influence to temperament than veterinarian respondents.

**Table 2:** Extent to which respondents believed each listed feature influenced sensitivity to pain in dogs. Results are shown for the general public and veterinary respondents. Results are the number of respondents in each category or response, and the percentage of those who rated that factor in parentheses. The highest response percentage for each factor is shown in bold.

		General Public					Veterinarians				
		N	Not at all	A little	Moderate amount	Great deal	N	Not at all	A little	Moderate amount	Great deal
Sensitivity to pain	Genetics	987	13 (1.3)	156 (15.8)	**433 (43.9)**	385 (39.0)	1053	14 (1.3)	196 (18.6)	**492 (46.7)**	351 (33.3)
	Environment they are raised in	989	46 (4.7)	207 (20.1)	339 (34.3)	**397 (40.1)**	1052	43 (29.5)	286 (27.2)	**413 (39.2)**	310 (29.5)
	Skin thickness	987	20 (2.0)	166 (16.8)	396 (40.1)	405 **(41.0)**	1046	162 (15.5)	**441 (42.1)**	346 (33.1)	97 (9.3)
	Temperament	981	24 (2.4)	185 (18.8)	**423 (43.1)**	349 (35.6)	1054	7 (0.7)	18 (1.7)	176 (16.7)	**853 (80.9)**
	Other	321	**156 (48.6)**	35 (10.9)	73 (22.7)	57 (17.8)	696	**308 (44.2)**	199 (28.6)	150 (21.6)	39 (5.6)

**Fig 2:**
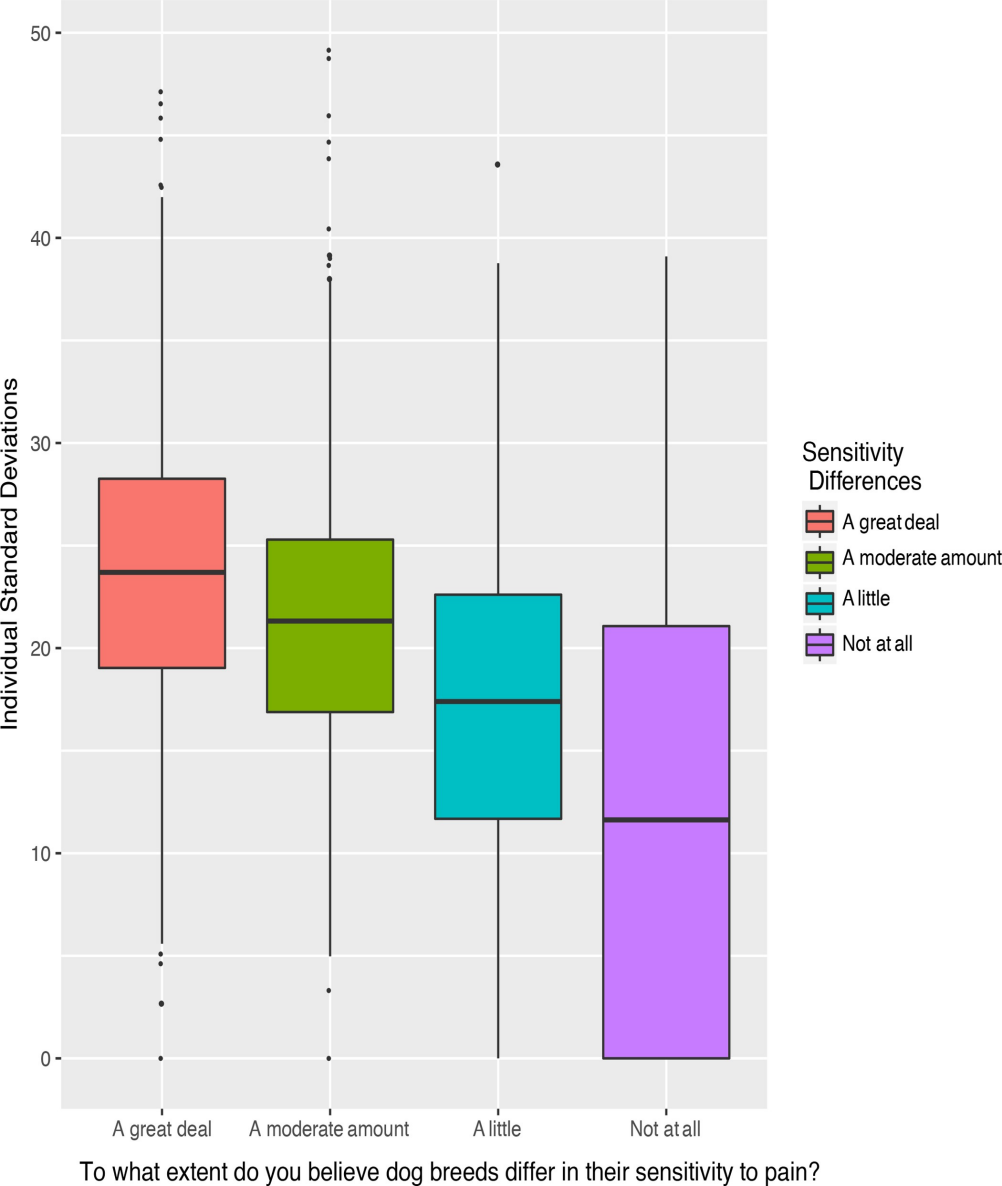
Box-and-whisker plot (median, interquartile range, and 1.5 interquartile range) of individual standard deviations across ratings of dog breeds for each level of response to the question regarding extent of belief in a difference in sensitivity for dog breeds. Standard deviations were significantly different for each group (corrected p < 0.05 for each comparison). This figure shows aggregate responses across the two groups; number of individuals in each group were: A great deal (n = 708), A moderate amount (n = 1007), A little (n = 335), and Not at all (n = 81).

As these responses were similar for “sensitivity to pain” and “response to pain,” the results for “sensitivity to pain” were considered for evaluating the association between strength of belief in a breed difference in sensitivity and standard deviation of an individual’s ratings. Bonferroni corrected Welch’s two-sample t-tests found significant differences between each level of response to the statement, “To what extent do you believe dog breeds differ in their sensitivity to pain?” and an individual respondent’s standard deviation of ratings across dog breeds. This finding was true for the general public, veterinarian, and aggregate responses (S5 Table in S1 File), with the exception that for the veterinarians, no significant difference was found for the “Not at all” and A little” responses when using a corrected p-value (full results in supplementary materials). The standard deviation of respondents’ ratings increased as the extent of belief (queried after all ratings had been made) increased from ‘none at all’ to ‘a great deal’ (Fig 2 and S1 Fig in S1 File).

#### Feelings thermometers and pain sensitivity ratings

The feelings thermometer asked respondents to rate how warm or cool they felt toward a breed or group on a scale from 0 (cool) to 100 (warm). These included four specific breeds (Golden retriever, Greyhound, Pitbull, and Pug) and groups (size [small/toy, medium/large, giant], pedigree [purebred, mixed breed], sex [male, female], and cats). For all breeds and size groups, significant relationships were found between the feelings thermometer ratings and pain sensitivity ratings. Positive values are interpreted as a higher feelings thermometer rating corresponding to a higher pain sensitivity rating, while negative values are interpreted as a higher feelings thermometer rating corresponding to a lower pain sensitivity rating. Results for each breed and size group are shown for both general public respondents and veterinarian respondents in Table 3. For each breed and size group, the direction of the relationship is the same; general public members have positive values for the relationships while veterinarians have negative values.

**Table 3:** Results of cumulative logit models of feelings thermometer ratings and pain sensitivity ratings for breeds and size groups. A Bonferroni-corrected significance level was set at α<0.0071. Uncorrected p-values are shown; all relationships were significant.

Breed/size group	General public		Veterinarian	
	z value	p-value	z value	p-value
Golden retriever	3.199	0.001	-7.568	3.782e^-14^
Greyhound	5.216	1.832 e^-7^	-5.087	3.630 e^-7^
Pug	8.341	7.343 e^-17^	-5.038	4.712 e^-7^
Pitbull	5.261	1.430 e^-7^	-4.344	1.398 e^-5^
Toy/small	15.285	9.573 e^-53^	-4.160	3.182 e^-5^
Medium/large	16.221	3.578 e^-59^	-4.360	1.300 e^-5^
Giant	5.965	2.447 e^-9^	-4.924	8.485 e^-7^

## Discussion

This study found that members of the general public and veterinarians rate dogs as having different pain sensitivity; ratings from general public respondents were driven mostly by size (with smaller dogs rated as more sensitive to pain) and also by a breed’s presence on breed specific legislative lists. Ratings from veterinarian respondents also fell along a spectrum of sensitivity ratings but were not as strongly related to size alone. Interestingly, the variance in the ratings by the veterinarians was low, with little difference between recent graduates and those with more experience with individual dog breeds. These findings provide strong support for the Generalized Group Characterization hypothesis, which predicts that dogs of different breeds will be rated as having different pain sensitivity based on group traits like physical appearance and social behavior stereotypes. Responses to each survey question were not required and it is unlikely that all general public respondents have first-hand knowledge or experience with all 28 breeds, particularly relative to the experience of pain. Despite this, the vast majority of respondents provided a rating for all the dogs. This suggests that rather than direct knowledge, people were willing to rate groups of dogs based on phenotype and reputation. For both groups, the extent to which respondents believe that dog breeds differ in their sensitivity and response to pain was high.

Among members of the general public, size (as an interaction between height and weight) appeared as the largest determinant of pain sensitivity ratings. Smaller, lighter dogs were rated as having higher pain sensitivity than larger, heavier dogs. Using breed-specific legislation as a proxy for social stereotypes toward dog breeds based on threat, this study found that a breed’s presence on breed-specific legislative lists was strongly associated with lower pain sensitivity ratings, even controlling for height, weight, and a height/weight interaction. These findings support the prediction that people rate pain sensitivity in dogs differently for different breeds based on their appearance and common group stereotypes (i.e. trustworthiness).

The ratings from veterinarians are important, as their perceptions could have consequences in the treatment of pain in dogs. While size appears to be an influential factor in pain sensitivity ratings among veterinarians, this is not as strong a relationship as in the general public responses. Indeed, size would not explain the high ratings for larger dogs, such as German Shepherd dog and Husky. Despite the absence of experimental investigation, breed differences in pain sensitivity or response have been suggested before in the veterinary literature [37, 38]. For example, in 1987, Crane refers to “breed-specific stoicism, such as that often attributed to the Pitbull terrier” [37]. Indeed, veterinarians rated the Pitbull significantly lower on the pain sensitivity scale than general public respondents. The lower variance in ratings among veterinary respondents also suggests that there are commonly held beliefs about different dog breeds.

For both groups, the extent to which respondents believe that dog breeds differ in their sensitivity and response to pain was high. In general, the strength of belief in individual factors that could affect pain sensitivity was similar between the general public and veterinarians. The exceptions were for temperament, which was credited with more influence on pain sensitivity and response to pain among the veterinarians than the general public, and skin thickness, which had the opposite relationship. While skin thickness does differ in different breeds of dogs, the relationship is not strictly driven by size and no correlation has been shown between skin thickness and body weight [39]. For example, the skin thickness of a Boxer is the same as a Great Dane, while a Yorkshire terrier’s is the same as a St. Bernard [39]. Thus, variation in skin thickness does not fully explain the differences in ratings shown here. In humans, differences in skin thickness has also been shown to be a false belief regarding bias in pain sensitivity ratings [15]. Both veterinarians and the general public respondents believed that genetics was an influential factor in pain sensitivity in dog breeds. Indeed, there is evidence that pain sensitivity is at least partially heritable [40], but this has not yet been demonstrated in dogs.

The strong effect of dog size may reflect a belief about a difference in the neurobiology of the pain system in smaller dogs, that they feel pain differently, or may indicate a belief in the relative ease of a given stimulus causing pain in a smaller dog. As an alternative explanation, evidence suggests that people have a slight preference for dogs with more paedomorphic features, often found in these smaller breeds, including larger eyes and wider-set eyes [41, 42]. These, and other features, have been described as *Kindchenschema* or baby schema—a collection of attributes found in infants across species that stimulate affection and lowered aggression (reviewed in [41]). It is possible that this baby schema affected pain sensitivity ratings by triggering increased empathy, a phenomenon found in studies comparing empathy ratings toward adults, children, and dogs [43, 44]. Further, commonly attributed behavioral signs of pain in animals include appearing “wide-eyed, anxious, nervous, trembling” or otherwise uncomfortable [38]; these signs may be more strongly associated with the smaller dogs depicted in this study.

The results of the feelings thermometers and their relationships to pain sensitivity ratings support a role for empathy for some respondents. For each breed and size group, there was a strong relationship between how warmly respondents felt toward that breed/group and their rating of that breed/group’s pain sensitivity. The positive relationship shown among the general public respondents is intuitive; the more warmly respondents felt toward a breed or group, the more sensitive to pain they rated them. A positive correlation has previously been shown between solidarity (i.e. feelings of psychological attachment and closeness) with animals and dispositional empathy, particularly with empathic concern [45]. While alternative explanations likely exist, warm feelings toward a breed or group may be related to solidarity, and higher pain sensitivity ratings to empathic concern. However, the results from the veterinarian sample are less easily interpreted. Among veterinarian respondents, the opposite relationship was found; the more warmly a respondent felt toward a breed or group, the *lower* they rated their pain sensitivity. It is possible that some or all veterinarian respondents interpreted pain sensitivity as behavioral reactivity, which can make handling more difficult in a health care setting. Thus, the more warmly one felt about a breed or group, the less sensitive/reactive (and therefore easier to handle) they believed that breed or group to be. Conversely, the less sensitive/reactive (and easier to handle) they believed the breed or group to be, the more warmly they felt toward them.

A limitation of the current study is that the scale does not differentiate between pain sensitivity as the actual perception of pain (a biological basis) and reactivity or response to painful stimuli (a behavioral characteristic). Critically, perspective on how a dog responds to pain versus how a dog perceives pain would be reasonably expected to drive recognition and treatment of pain. Given this scale (not at all sensitive to most sensitive imaginable) has been used to evaluate pain sensitivity bias in people [18], this was a rational starting point for dogs. We present strong evidence that people rate pain sensitivity differently based on breed-specific stereotypes or phenotypic traits and dog breed archetypes. Given the propensity of humans to categorize by trait, the Generalized Group Characterization hypothesis would predict similar patterns toward other non-human companion animal species that exhibit a range of phenotypic types and associated stereotypes (e.g. horses and cats). This study has identified a phenomenon; further investigation is needed to more fully comprehend the impact.

## Supporting information

S1 File (DOCX)

## References

[1] Hare B WV. Survival of the Friendliness: Random House; (in press).

[2] Avenanti A, Sirigu A, Aglioti SM. Racial bias reduces empathic sensorimotor resonance with other-race pain. Curr Biol. 2010;20(11):1018–22. 10.1016/j.cub.2010.03.071 20537539

[3] Kteily N, Bruneau E. Backlash: The Politics and Real-World Consequences of Minority Group Dehumanization. Pers Soc Psychol Bull. 2017;43(1):87–104. 10.1177/0146167216675334 28903649

[4] Burgess DJ, van Ryn M, Crowley-Matoka M, Malat J. Understanding the provider contribution to race/ethnicity disparities in pain treatment: Insights from dual process models of stereotyping. Pain Med. 2006;7(2):119–34. 10.1111/j.1526-4637.2006.00105.x 16634725

[5] Green CR, Anderson KO, Baker TA, Campbell LC, Decker S, Fillingim RB, et al The unequal burden of pain: confronting racial and ethnic disparities in pain. Pain Med. 2003;4(3):277–94. 10.1046/j.1526-4637.2003.03034.x 12974827

[6] Qiao WP, Powell ES, Witte MP, Zelder MR. Relationship between racial disparities in ED wait times and illness severity. Am J Emerg Med. 2016;34(1):10–5. 10.1016/j.ajem.2015.08.052 26454472

[7] Park CY, Lee MA, Epstein AJ. Variation in Emergency Department Wait Times for Children by Race/Ethnicity and Payment Source. Health Serv Res. 2009;44(6):2022–39. 10.1111/j.1475-6773.2009.01020.x 19732167 PMC2796312

[8] Schafer G, Prkachin KM, Kaseweter KA, Williams ACD. Health care providers' judgments in chronic pain: the influence of gender and trustworthiness. Pain. 2016;157(8):1618–25. 10.1097/j.pain.0000000000000536 26934512

[9] Hollingshead NA, Matthias MS, Bair MJ, Hirsh AT. Impact of Race and Sex on Pain Management by Medical Trainees: A Mixed Methods Pilot Study of Decision Making and Awareness of Influence. Pain Med. 2015;16(2):280–90. 10.1111/pme.12506 25039974

[10] Uri O, Elias S, Behrbalk E, Halpern P. No gender-related bias in acute musculoskeletal pain management in the emergency department. Emerg Med J. 2015;32(2):149–52. 10.1136/emermed-2013-202716 24123168

[11] Burgess DJ, Fu SS, van Ryn M. Why do providers contribute to disparities and what can be done about it? J Gen Intern Med. 2004;19(11):1154–9. 10.1111/j.1525-1497.2004.30227.x 15566446 PMC1494785

[12] Cintron A, Morrison RS. Pain and ethnicity in the United States: A systematic review. Journal of Palliative Medicine. 2006;9(6):1454–73. 10.1089/jpm.2006.9.1454 17187552

[13] Sabin JA, Greenwald AG. The influence of implicit bias on treatment recommendations for 4 common pediatric conditions: pain, urinary tract infection, attention deficit hyperactivity disorder, and asthma. Am J Public Health. 2012;102(5):988–95. 10.2105/AJPH.2011.300621 22420817 PMC3483921

[14] Dore RA, Hoffman KM, Lillard AS, Trawalter S. Children's racial bias in perceptions of others' pain. Brit J Dev Psychol. 2014;32(2):218–31. 24576067 10.1111/bjdp.12038

[15] Hoffman KM, Trawalter S, Axt JR, Oliver MN. Racial bias in pain assessment and treatment recommendations, and false beliefs about biological differences between blacks and whites. P Natl Acad Sci USA. 2016;113(16):4296–301. 10.1073/pnas.1516047113 PMC4843483 27044069

[16] Trawalter S, Hoffman KM. Got Pain? Racial Bias in Perceptions of Pain. Soc Personal Psychol. 2015;9(3):146–57.

[17] Trawalter S, Hoffman KM, Waytz A. Racial Bias in Perceptions of Others' Pain. Plos One. 2012;7(11). 10.1371/journal.pone.0048546 PMC3498378 23155390

[18] Hollingshead NA, Meints SM, Miller MM, Robinson ME, Hirsh AT. A comparison of race-related pain stereotypes held by White and Black individuals. J Appl Soc Psychol. 2016;46(12):718–23. 10.1111/jasp.12415 28496282 PMC5421992

[19] Lockwood PL. The anatomy of empathy: Vicarious experience and disorders of social cognition. Behav Brain Res. 2016;311:255–66. 10.1016/j.bbr.2016.05.048 27235714 PMC4942880

[20] Sessa P, Meconi F. Perceived trustworthiness shapes neural empathic responses toward others' pain. Neuropsychologia. 2015;79:97–105. 10.1016/j.neuropsychologia.2015.10.028 26514617

[21] Cikara M, Bruneau EG, Saxe RR. Us and Them: Intergroup Failures of Empathy. Curr Dir Psychol. 2011;20(3):149–53.

[22] Prguda E, Neumann DL. Inter-human and animal-directed empathy: a test for evolutionary biases in empathetic responding. Behav Processes. 2014;108:80–6. 10.1016/j.beproc.2014.09.012 25242725

[23] Norring M, Wikman I, Hokkanen AH, Kujala MV, Hanninen L. Empathic veterinarians score cattle pain higher. Vet J. 2014;200(1):186–90. 10.1016/j.tvjl.2014.02.005 24685101

[24] Ellingsen K, Zanella AJ, Bjerkas E, Indrebo A. The Relationship between Empathy, Perception of Pain and Attitudes toward Pets among Norwegian Dog Owners. Anthrozoos. 2010;23(3):231–43.

[25] Klinck MP, Mogil JS, Moreau M, Lascelles BDX, Flecknell PA, Poitte T, et al Translational pain assessment: could natural animal models be the missing link? Pain. 2017;158(9):1633–46. 10.1097/j.pain.0000000000000978 28614187

[26] Irvine L, Cilia L. More-than-human families: Pets, people, and practices in multispecies households. Sociol Compass. 2017;11(2):13.

[27] Meeson RL, Todhunter RJ, Blunn G, Nuki G, Pitsillides AA. Spontaneous dog osteoarthritis—a One Medicine vision. Nat Rev Rheumatol. 2019;15(5):273–87. 10.1038/s41584-019-0202-1 30953036 PMC7097182

[28] Sevillano V, Fiske ST. Warmth and competence in animals. J Appl Soc Psychol. 2016;46(5):276–93.

[29] Protopopova A, Wynne CDL. Judging a Dog by Its Cover: Morphology but Not Training Influences Visitor Behavior toward Kenneled Dogs at Animal Shelters. Anthrozoos. 2016;29(3):469–87.

[30] Sinski J, Carini RM, Weber JD. Putting (Big) Black Dog Syndrome to the Test: Evidence from a Large Metropolitan Shelter. Anthrozoos. 2016;29(4):639–52.

[31] Posage JM, Bartlett PC, Thomas DK. Determining factors for successful adoption of dogs from an animal shelter. J Am Vet Med Assoc. 1998;213(4):478–82. 9713528

[32] Strand P, New J, Smith FO, Reichman B. 217 Outdated perceptions influence the acquisition of pet dogs in the United States and quietly reshape the dog market place. Journal of Animal Science. 2017;95(supplement4):107–8.

[33] Schoenebeck JJ, Ostrander EA. Insights into Morphology and Disease from the Dog Genome Project In: Schekman R, Lehmann R, editors. Annual Review of Cell and Developmental Biology, Vol 30. Annual Review of Cell and Developmental Biology. 30 Palo Alto: Annual Reviews; 2014 p. 535–60. 10.1146/annurev-cellbio-100913-012927 PMC5510473 25062362

[34] Knazovicky D, Helgeson ES, Case B, Thomson A, Gruen ME, Maixner W, et al Replicate effects and test-retest reliability of quantitative sensory threshold testing in dogs with and without chronic pain. Vet Anaesth Analg. 2017;44(3):615–24. 10.1016/j.vaa.2016.08.008 28528759

[35] Sevillano V, Fiske ST. Animals as Social Objects Groups, Stereotypes, and Intergroup Threats. European Psychologist. 2016;21(3):206–17.

[36] Parker HG, Dreger DL, Rimbault M, Davis BW, Mullen AB, Carpintero-Ramirez G, et al Genomic Analyses Reveal the Influence of Geographic Origin, Migration, and Hybridization on Modern Dog Breed Development. Cell Rep. 2017;19(4):697–708. 10.1016/j.celrep.2017.03.079 28445722 PMC5492993

[37] Crane SW. Perioperative analgesia: a surgeon's perspective. J Am Vet Med Assoc. 1987;191(10):1254–7. 3692963

[38] Haskins SC. Use of analgesics postoperatively and in a small animal intensive care setting. J Am Vet Med Assoc. 1987;191(10):1266–8. 3692966

[39] Diana A, Preziosi R, Guglielmini C, Degliesposti P, Pietra M, Cipone M. High-frequency ultrasonography of the skin of clinically normal dogs. Am J Vet Res. 2004;65(12):1625–30. 10.2460/ajvr.2004.65.1625 15631025

[40] Mogil JS, Wilson SG, Bon K, Lee SE, Chung K, Raber P, et al Heritability of nociception II. 'Types' of nociception revealed by genetic correlation analysis. Pain. 1999;80(1–2):83–93. 10.1016/s0304-3959(98)00196-1 10204720

[41] Hecht J, Horowitz A. Seeing Dogs: Human Preferences for Dog Physical Attributes. Anthrozoos. 2015;28(1):153–63.

[42] Kaminski J, Waller BM, Diogo R, Hartstone-Rose A, Burrows AM. Evolution of facial muscle anatomy in dogs. Proc Natl Acad Sci U S A. 2019;116(29):14677–81. 10.1073/pnas.1820653116 31209036 PMC6642381

[43] Batson CD, Lishner DA, Cook J, Sawyer S. Similarity and nurturance: Two possible sources of empathy for strangers. Basic Appl Soc Psychol. 2005;27(1):15–25.

[44] Levin J, Arluke A, Irvine L. Are People More Disturbed by Dog or Human Suffering? Influence of Victim's Species and Age. Soc Anim. 2017;25(1):1–16.

[45] Amiot CE, Bastian B. Solidarity with Animals: Assessing a Relevant Dimension of Social Identification with Animals. Plos One. 2017;12(1). 10.1371/journal.pone.0168184 PMC5207407 28045909

