# Supplementary material for: Do dog breeds differ in pain sensitivity? Veterinarians and the public believe they do
Source: PLoS One. 2020 Mar 17;15(3):e0230315. doi: 10.1371/journal.pone.0230315 (PMC7077843; doi:10.1371/journal.pone.0230315)

Supplementary Materials:

**Methods**

*Survey construction and pilot evaluation*

**General public version**

The survey instrument was composed using standardized survey software (Qualtrics®), and two forms of the survey (A and B) were constructed. Twenty-eight dog breeds were selected to encompass a range of sizes, body and head shapes, and coat types/lengths and shown in Table S1. For six of the breeds, pictures were selected with two different coat colors (both colors were acceptable as part of the breed standard). One of each of these pairs was randomly assigned to form A, with the other assigned to form B. This meant that in the final survey, 22/28 (79%) dog breeds were identical across the forms, while 6/28 (21%) were shown in one of two possible colors.

Table S1. Dog breeds included in the survey. For the six dog breeds shown as two color variants, colors used on forms A and B are indicated. Those breeds that have been included in breed-specific legislative (BSL) lists are shown with an asterisk (*).

| Border Collie | Dachshund (A: tan; B: black/tan) | Husky | Pug |
| --- | --- | --- | --- |
| Boston Terrier | Doberman Pinscher* | Jack Russel Terrier | Rhodesian Ridgeback |
| Boxer | German Shepherd dog | Labrador retriever | Rottweiler* |
| Bulldog (English) | Golden retriever | Maltese | Samoyed |
| Cavalier King Charles Spaniel | Gordon Setter | English Mastiff* | Schnauzer (A: white; B: black and silver) |
| Chihuahua (A: black/tan; B: tan) | Great Dane (A: Black; B: Black/white) | Pit Bull (American Stafforshire Terrier)* | Weimaraner |
| Chow Chow (A: cinnamon; B: black) | Greyhound | Pomeranian (A: black/tan; B: tan) | Whippet |

Following survey construction, a pilot with 100 respondents was conducted using Amazon’s Mechanical Turk (MTurk) service. Respondents were asked to complete the survey and provide feedback, and were compensated $0.75 for their time. This pilot was used to ensure that the survey was easy to comprehend and flowed correctly. It also provided a way to estimate time for completion. None of the data collected in this pilot were included in the analyses. Following this pilot, small changes were made in wording to improve clarity, and no further changes were made thereafter.

**Results**

**General public survey**

A total of 1063 surveys were returned. Eight survey responses were excluded following review of the feedback (seven who detailed confusion with the subject of the question or format, and one who was unable to see any of the pictures so rated all dogs the same). After removing gender non-binary responses (n = 2), there were 1053 survey responses entered into the analyses, with form A having 521 responses and form B having 532 responses.

Demographic characteristics were similar for respondents of forms A and B (Table S2). No significant differences were found between respondents of forms A and B in distribution of gender or age, education level (Chi-sq. 4.753, p = 0.447), race (Chi-sq. 0.199, p = 0.225; 65.6% Caucasian, 12.4% African American/black/Caribbean, 11.2% Hispanic), or amount of experience with dogs (Chi-sq. 0.650, p = 0.885; 10.2% never owned a dog, 25.4% had some experience, 55.6% had moderate experience, and 8.7% had a high amount of experience).

Table S2. Gender and age distribution analysis for general public respondents on survey forms A and B.

|  | Gender | | | | Age category | | | | | | | |
| --- | --- | --- | --- | --- | --- | --- | --- | --- | --- | --- | --- | --- |
|  | M | F | Chi-sq. | p-value | 18-22 | 23-30 | 31-40 | 41-50 | 51-60 | >60 | Chi- sq. | p-value |
| Form A | 256 | 265 | 0.007 | 0.951 | 65 | 112 | 105 | 82 | 85 | 72 | 6.38 | 0.271 |
| Form B | 260 | 272 |  |  | 63 | 88 | 113 | 106 | 91 | 71 |  |  |

*Pain sensitivity ratings for dog breeds*

Table S3: Difference is in the Log-odds (higher sensitivity / lower or equal sensitivity) between forms A and B for the six breeds that differed between the two forms. The two breeds whose confidence intervals (CIs) do not include zero are in bold.

| Breed | Difference (form B – form A) | 95% CI lower bound | 95% CI upper bound |
| --- | --- | --- | --- |
| **Chihuahua** | **0.93** | **0.71** | **1.14** |
| Chow Chow | -0.07 | -0.27 | 0.14 |
| Great Dane | 0.18 | -0.02 | 0.39 |
| Dachshund | 0.03 | -0.19 | 0.24 |
| **Pomeranian** | **0.64** | **0.42** | **0.85** |
| Schnauzer | 0.01 | -0.19 | 0.21 |

*Pain sensitivity related to participant and dog variables*

In the final model, significant effects were found for self-pain rating (z = 10.58, p < 0.0001) with higher self-pain rating associated with higher pain sensitivity ratings for dogs, age 18-22 (baseline age = >60, z = -2.04, p = 0.041) with the youngest age group rating breeds as less sensitive to pain overall, and other race (majority Hispanic; baseline race = Caucasian, z = -2.81, p = 0.005), such that identifying as a race other than Caucasian or African-American was associated with lower pain sensitivity ratings for dogs overall. No significant effects were found for education or dog experience.

**Veterinarian survey**

A total of 1,237 responses were collected. All respondents outside North America were excluded, as international differences between dog breed distribution could affect responses. After excluding cases with no gender response (n=22), this left a total of 1078 responses used in the analyses.

**Comparisons between the general public and veterinarian surveys**

*Beliefs regarding differences in pain sensitivity and reaction to pain*

Table S4. Extent to which respondents believed each listed feature influenced response to pain in dogs. Results are shown for the general public and veterinary respondents. Results are the number of respondents in each category or response, and the percentage of those that rated that factor in parentheses. The highest response percentage for each factor is shown in bold.

| General Public | | | | | | |  | Veterinarians | | | | |
| --- | --- | --- | --- | --- | --- | --- | --- | --- | --- | --- | --- | --- |
| Response to pain | Genetics | 994 | 13 (1.3) | 155 (15.6) | **426 (42.9)** | 400 (40.2) |  | 1070 | 26 (2.4) | 186 (17.5) | **482 (45.0)** | 376 (35.1) |
|  | Environment they are raised in | 993 | 37 (3.7) | 189 (19.0) | 356 (35.9) | **411 (41.4)** |  | 1065 | 33 (3.1) | 186 (17.4) | 394 (37.0) | **452 (42.4)** |
|  | Skin thickness | 991 | 24 (2.4) | 172 (17.4) | **436 (44.0)** | 359 (36.2) |  | 1060 | 195 (18.4) | **465 (43.9)** | 333 (31.4) | 67 (6.3) |
|  | Temperament | 991 | 16 (1.6) | 172 (17.4) | **429 (43.3)** | 374 (37.7) |  | 1068 | 4 (0.4) | 19 (1.8) | 185 (17.3) | **860 (80.5)** |
|  | Other | 335 | **156 (46.6)** | 43 (12.8) | 81 (24.2) | 55 (16.4) |  | 630 | **394 (62.5)** | 111 (17.6) | 84 (13.3) | 41 (6.5) |

The factor “Other” was selected at any level (a little, or above) by both general public and veterinarian respondents. Categorization of free-text responses found that among the general public responses (n=58), only one category had >10% of these respondents, and this was size (n=20). Among the veterinary responses (n=344), comments indicated that some respondents, while making breed level sensitivity ratings, made individual level judgements about factors, with >10% of these respondents indicating owner effects (n=87), individual temperament (n=42), fear/stress/anxiety (n=44), and context/situation (n=55).

Bonferroni corrected Welch’s two-sample t-tests found significant differences between each almost each level of response to the statement, “To what extent do you believe dog breeds differ in their sensitivity to pain?” and an individual respondent’s standard deviation of ratings across dog breeds. This finding was true for the general public, veterinarian, and aggregate responses (Table S5), with the exception that for the veterinarians, no significant difference was found for the “Not at all” and A little” responses when using a corrected p-value. The standard deviation of respondents’ ratings increased as the extent of belief (queried after all ratings had been made) increased from ‘none at all’ to ‘a great deal’ (Fig S1).

Table S5. Results of Bonferroni corrected Welch’s two-sample t-tests comparing each level of response to the statement, “To what extent do you believe dog breeds differ in their sensitivity to pain?” and an individual respondent’s standard deviation of ratings across dog breeds. Results are shown for general public, veterinarian, and aggregate responses.

| Aggregate Responses |  | A great deal | A moderate amount | A little |
| --- | --- | --- | --- | --- |
|  | Moderate | t = 6.53  p = 5.41e-10  df = 1440.70 |  |  |
|  | A little | t = 12.48  p < 2.2e-16  df = 612.33 | t = 8.56  p < 2.2e-16  df = 505.07 |  |
|  | Not at all | t = 8.54  p = 2.43e-10  df = 86.31 | t = 6.89  p = 5.99e-09  df = 83.32 | t = 3.59  p = 0.0312  df = 97.37 |
| General public | Moderate | t = 2.96  p = 2.06e-02  df = 608.59 |  |  |
|  | A little | t = 8.92  p = 2.72e-18  df = 423.76 | t = 7.43  p = 5.32e-12  df = 328.37 |  |
|  | Not at all | t = 7.25  p = 8.34e-21  df = 76.35 | t = 6.27  p = 5.66e-16  df = 68.96 | t = 2.72  p = 2.20e-03  df = 83.62 |
| Veterinarian | Moderate | t = 7.26  p = 5.41e-12  df = 848.27 |  |  |
|  | A little | t = 8.61  p = 9.06e-15  df = 217.15 | t = 4.29  p = 1.64e-04  df = 199.34 |  |
|  | Not at all | t = 4.39  p = 2.56e-03  df = 16.57 | t = 3.12  p = 3.90e-02  df = 16.44 | t = 1.85  p = 0.48  df = 18.21 |

Fig S1. Box-and-whisker plot (median, interquartile range, and 1.5 interquartile range) of individual standard deviations across ratings of dog breeds for each level of response to the question regarding extent of belief in a difference in sensitivity for dog breeds. Standard deviations were significantly different for each group (corrected p < 0.05 for each comparison). This figure shows separate responses for the general public and veterinarian respondents.

Supplementary Material: S3 **Survey**


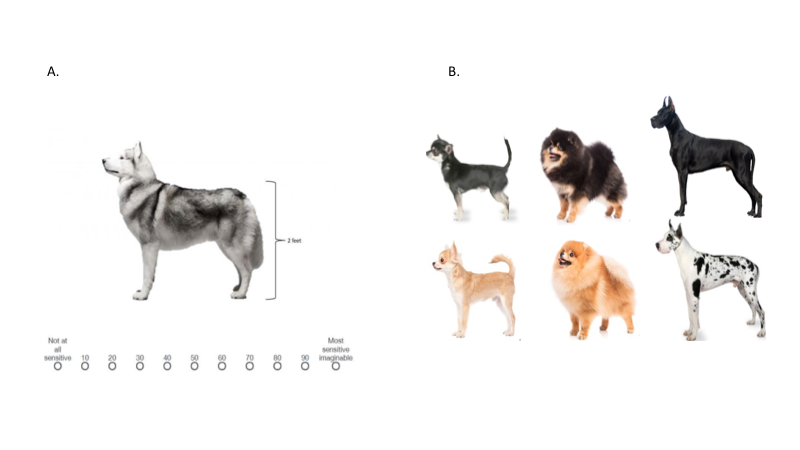


Fig S1. (A) Example of a dog photo and scale subjects were invited to use; (B) Pictures shown in forms A and B of the general public survey for Chihuahua, Pomeranian, and Great Dane. Great Danes not shown to scale.

Figure S2. Survey (Form B)


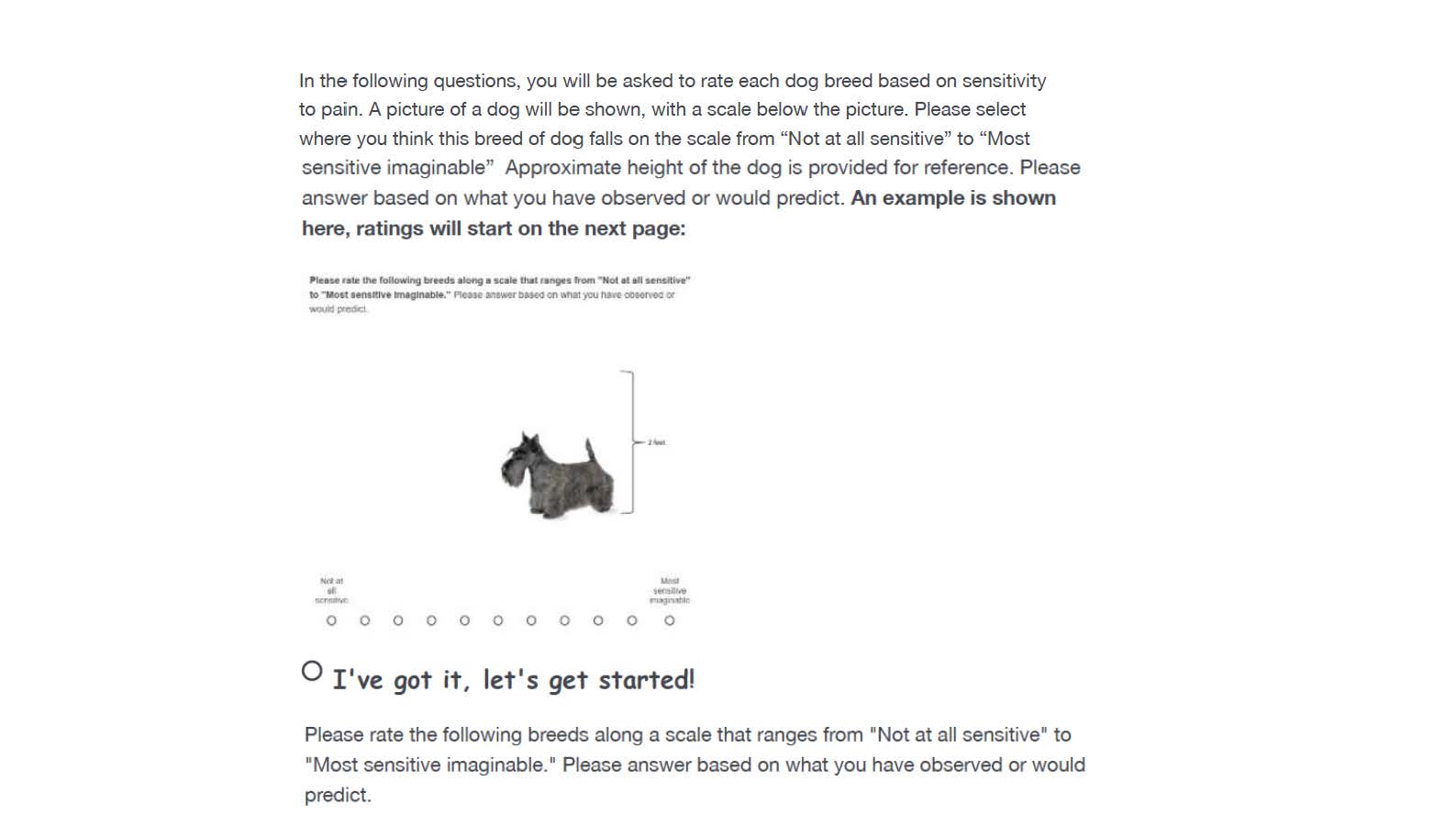


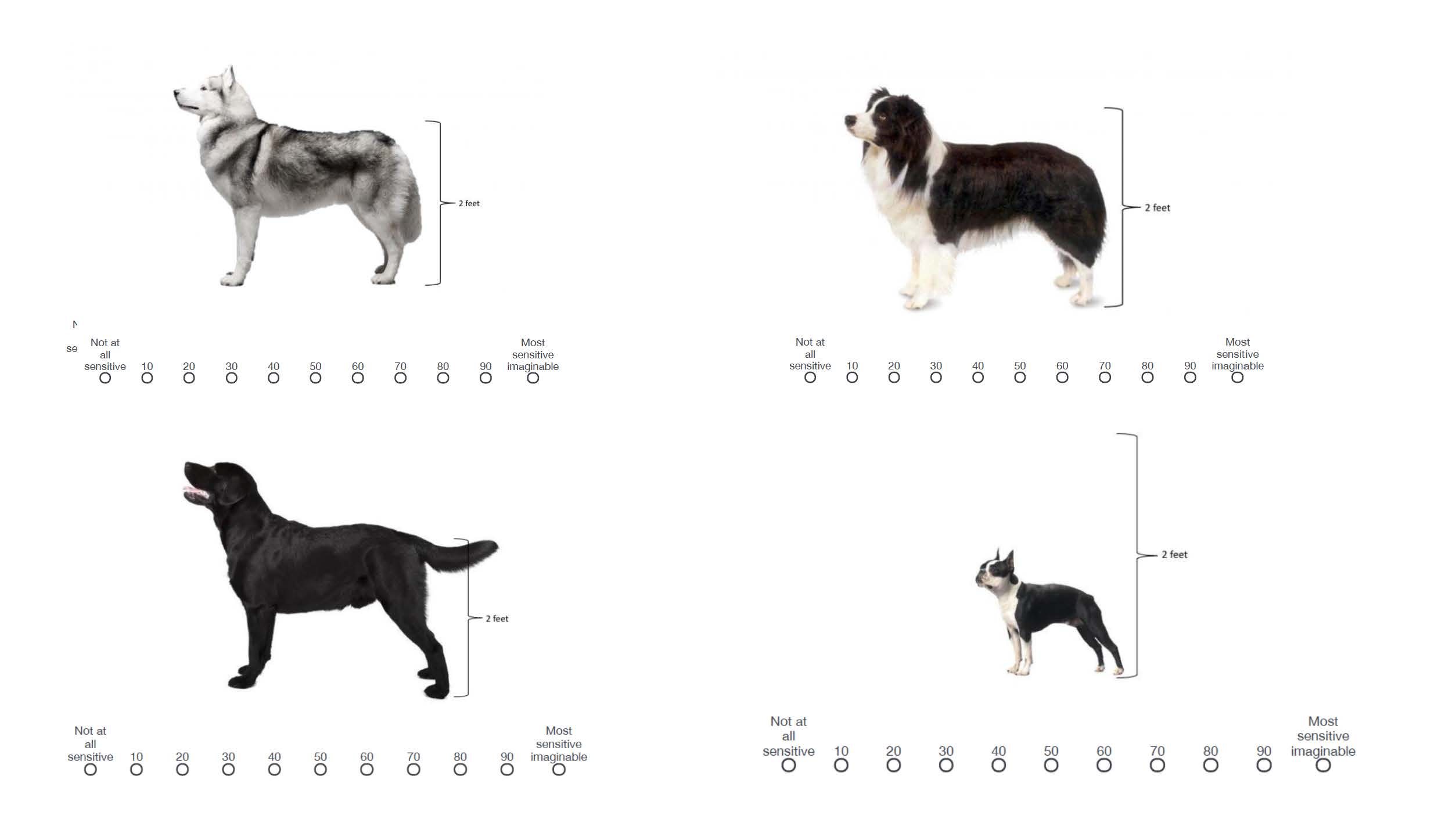


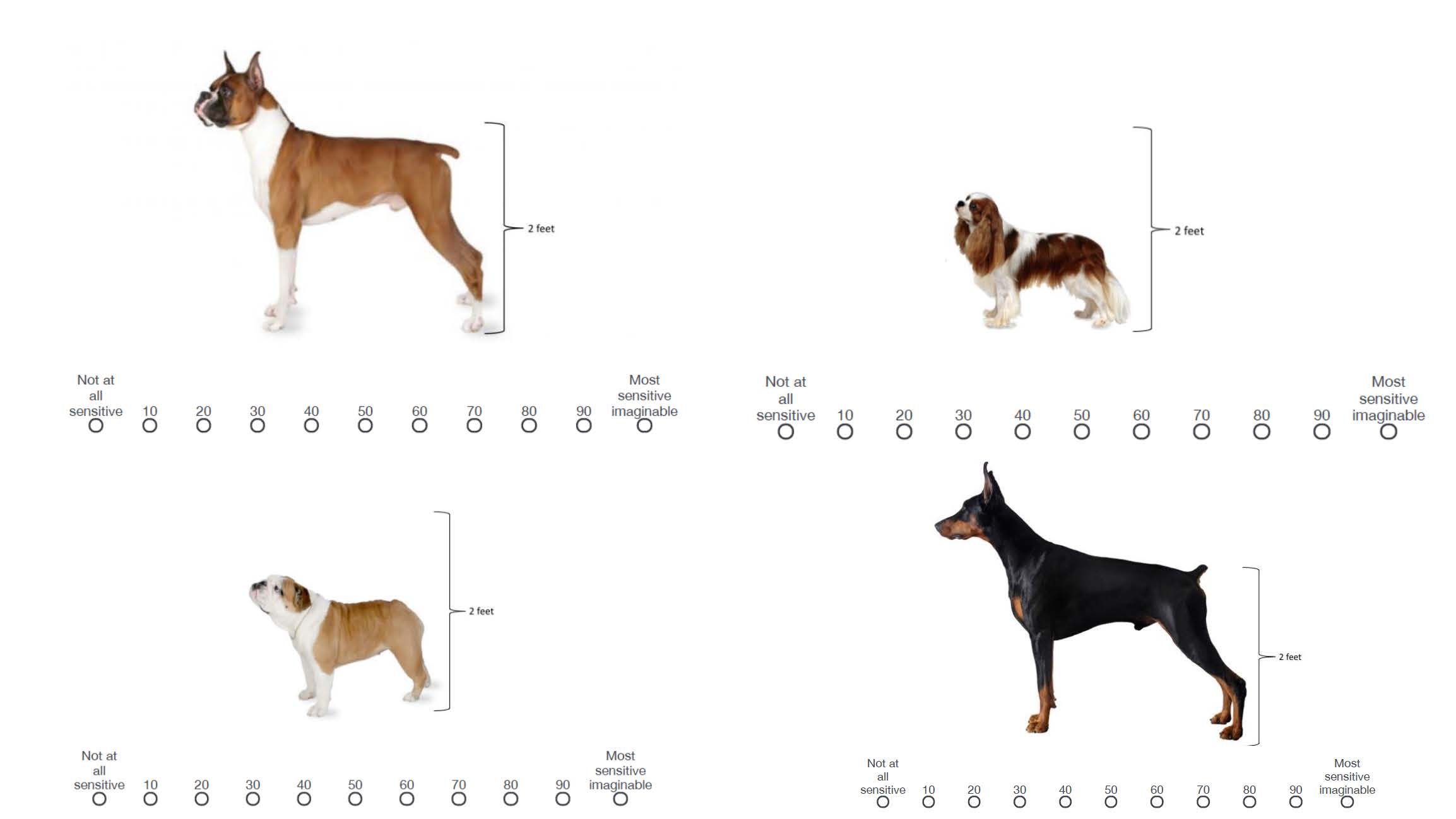


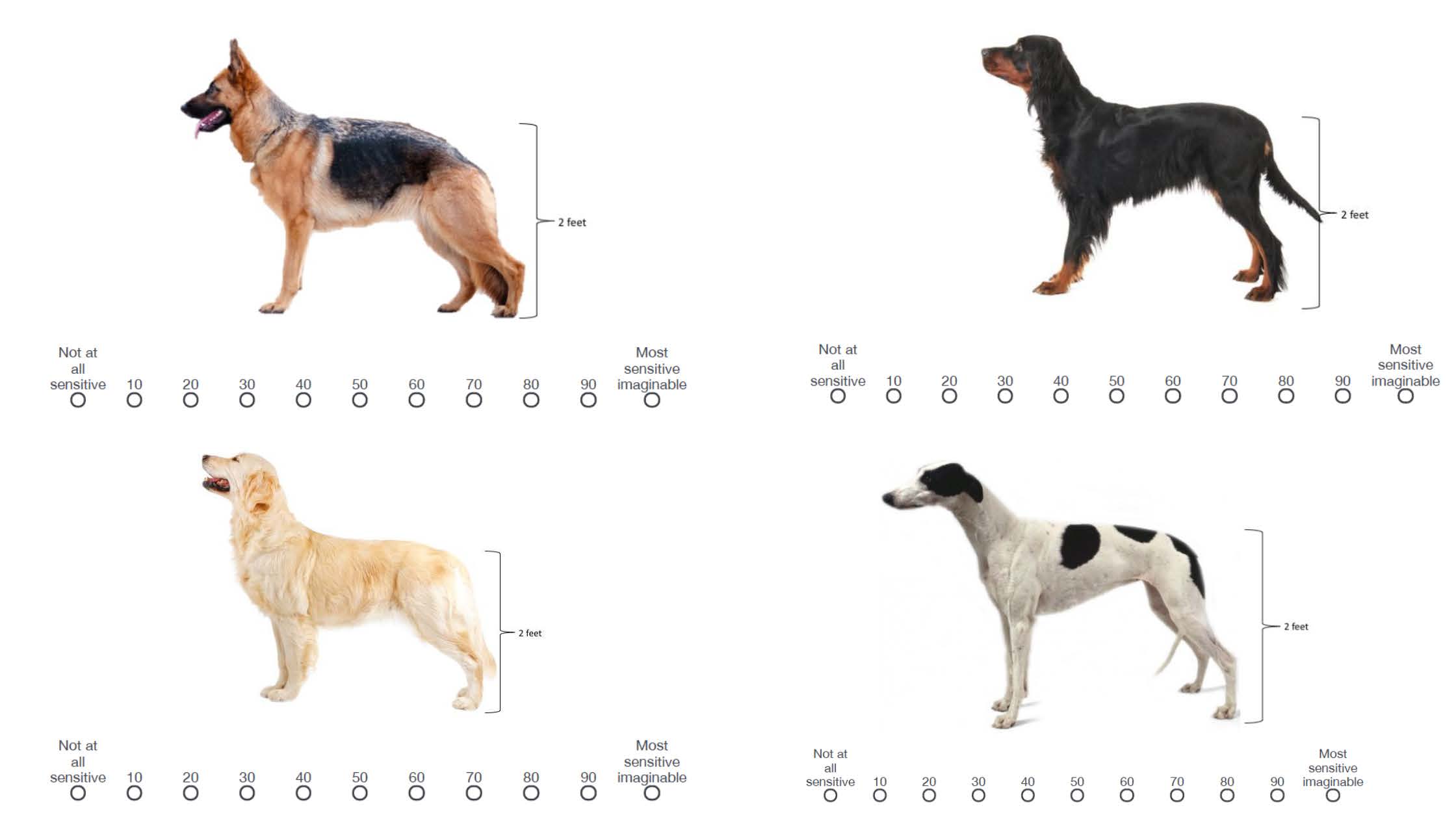


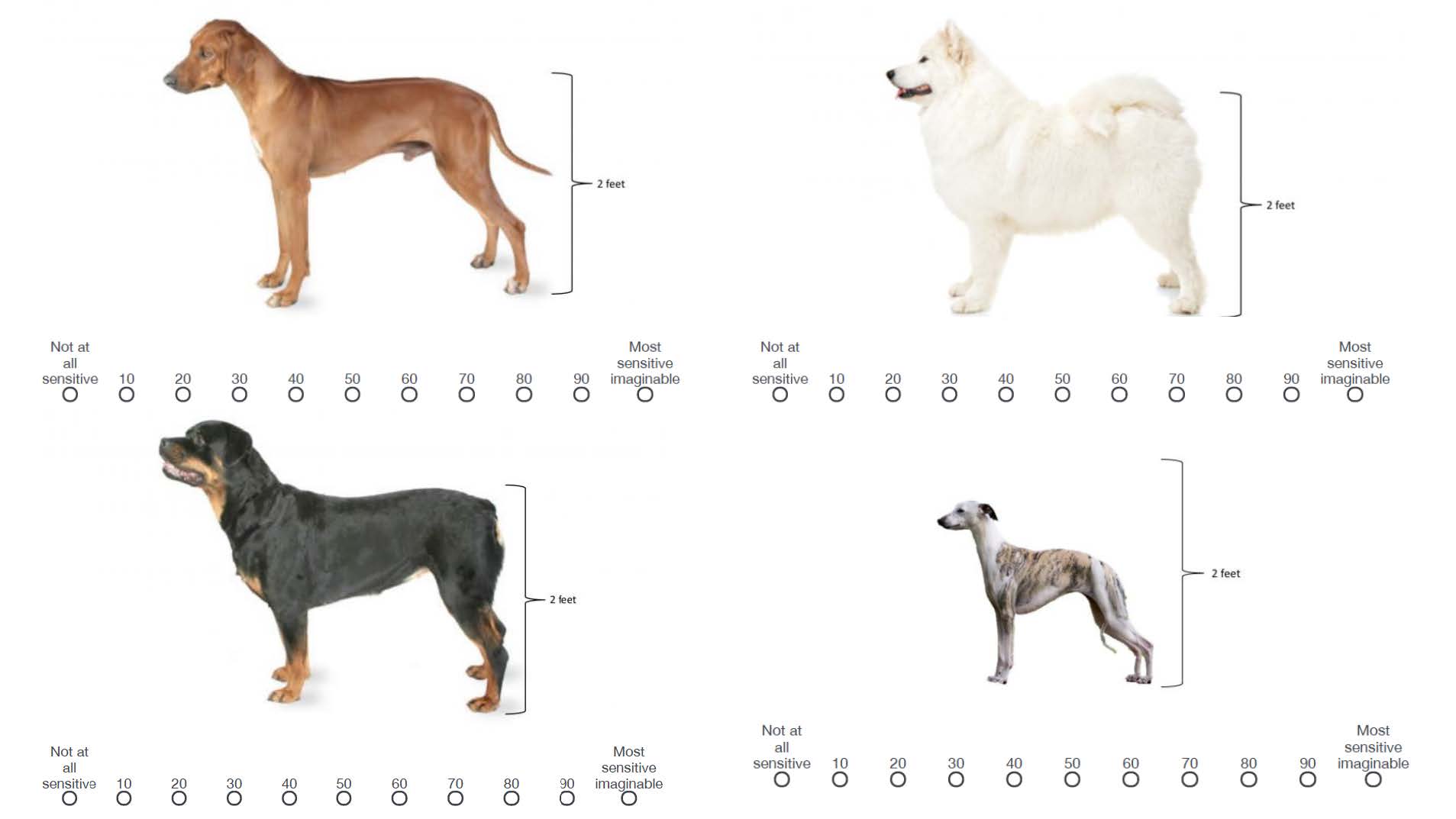


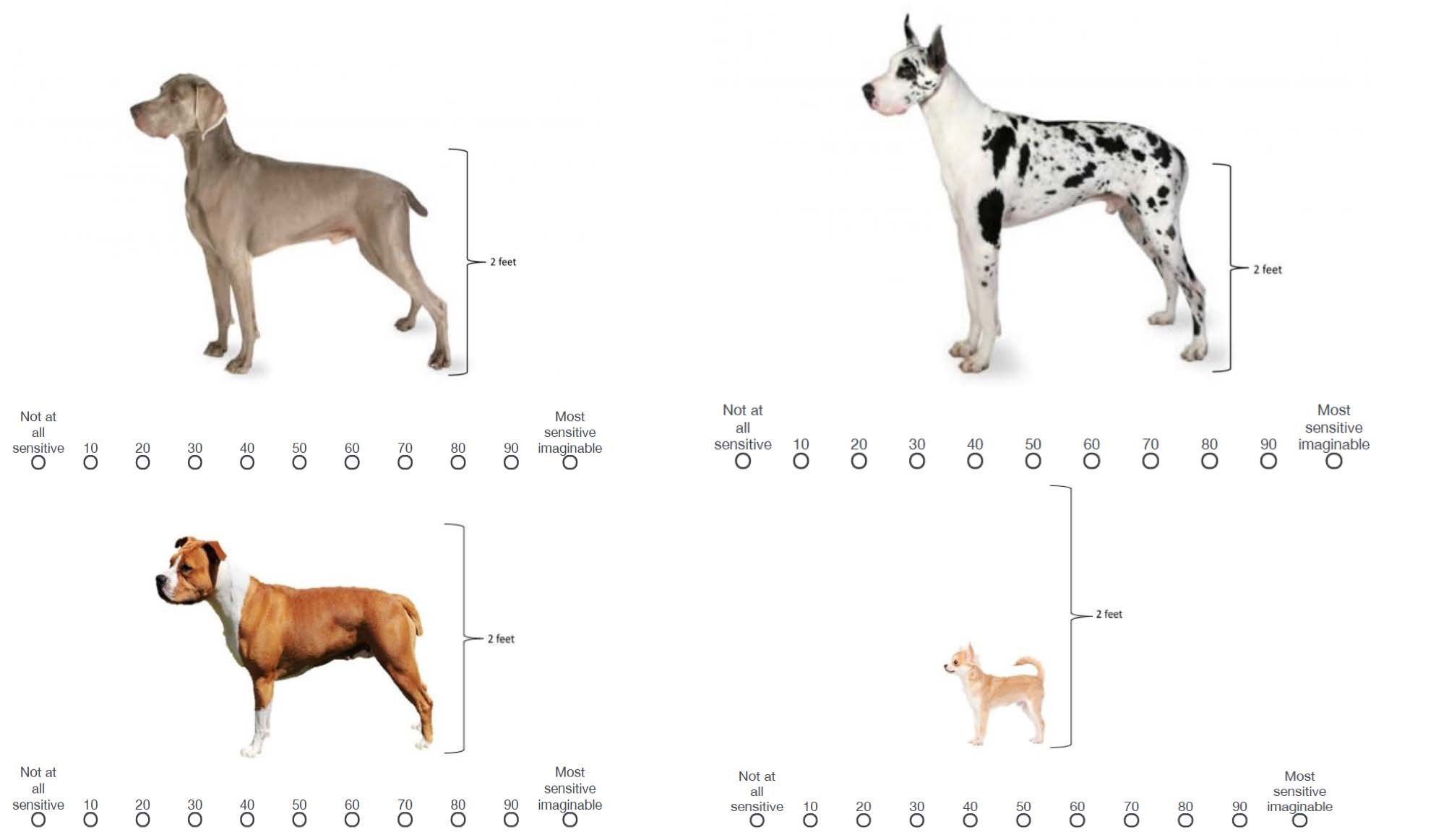


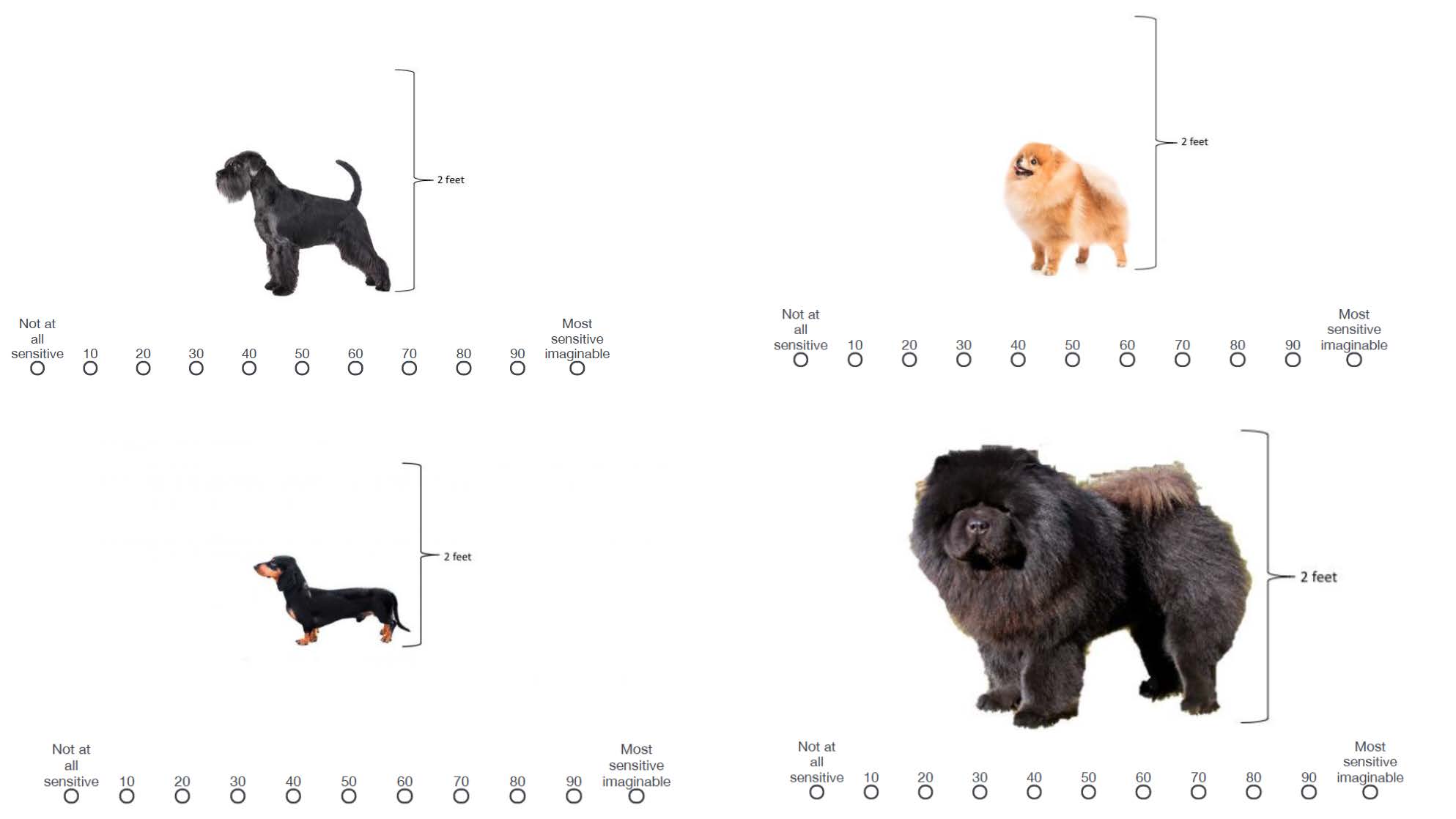


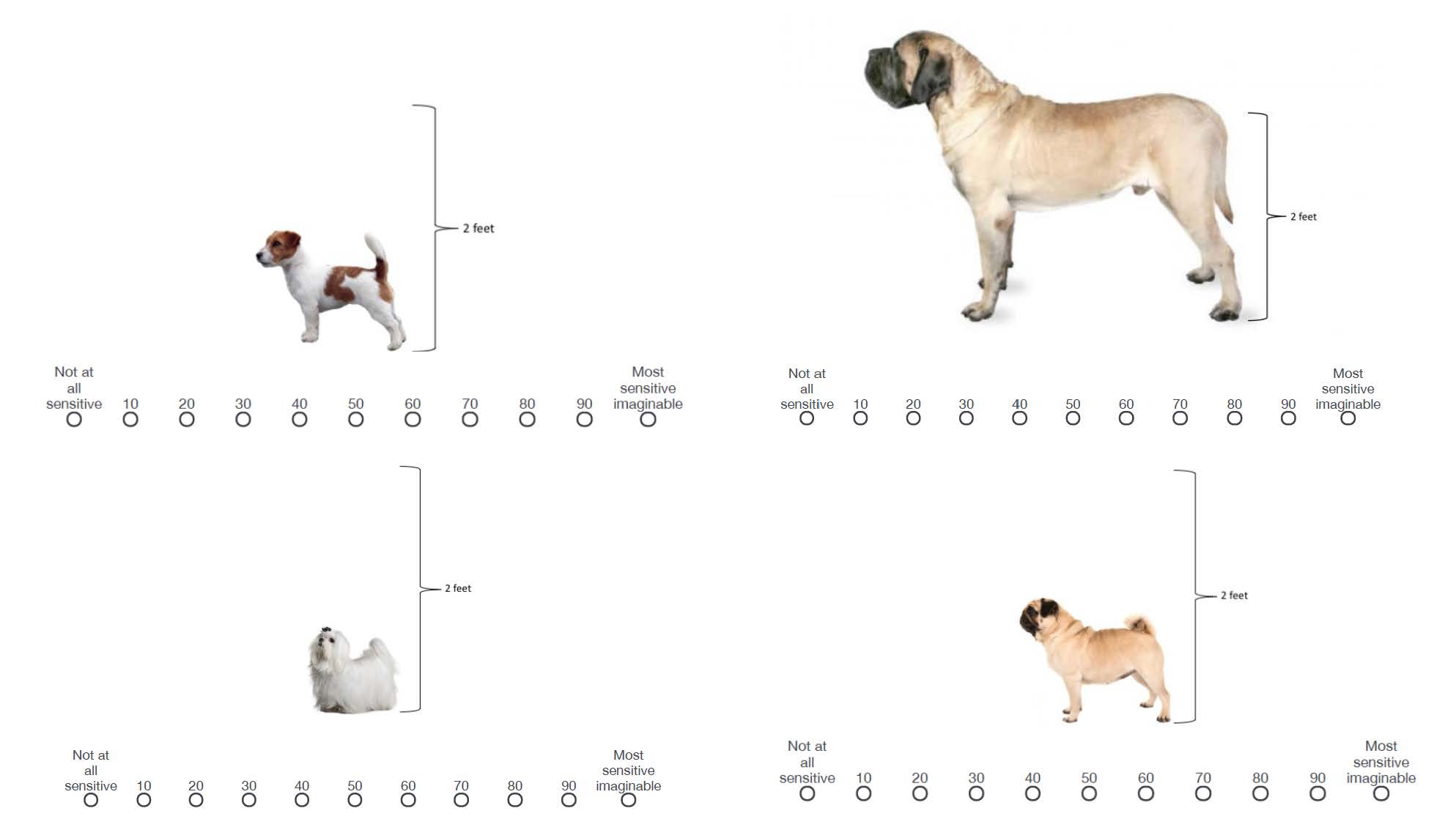


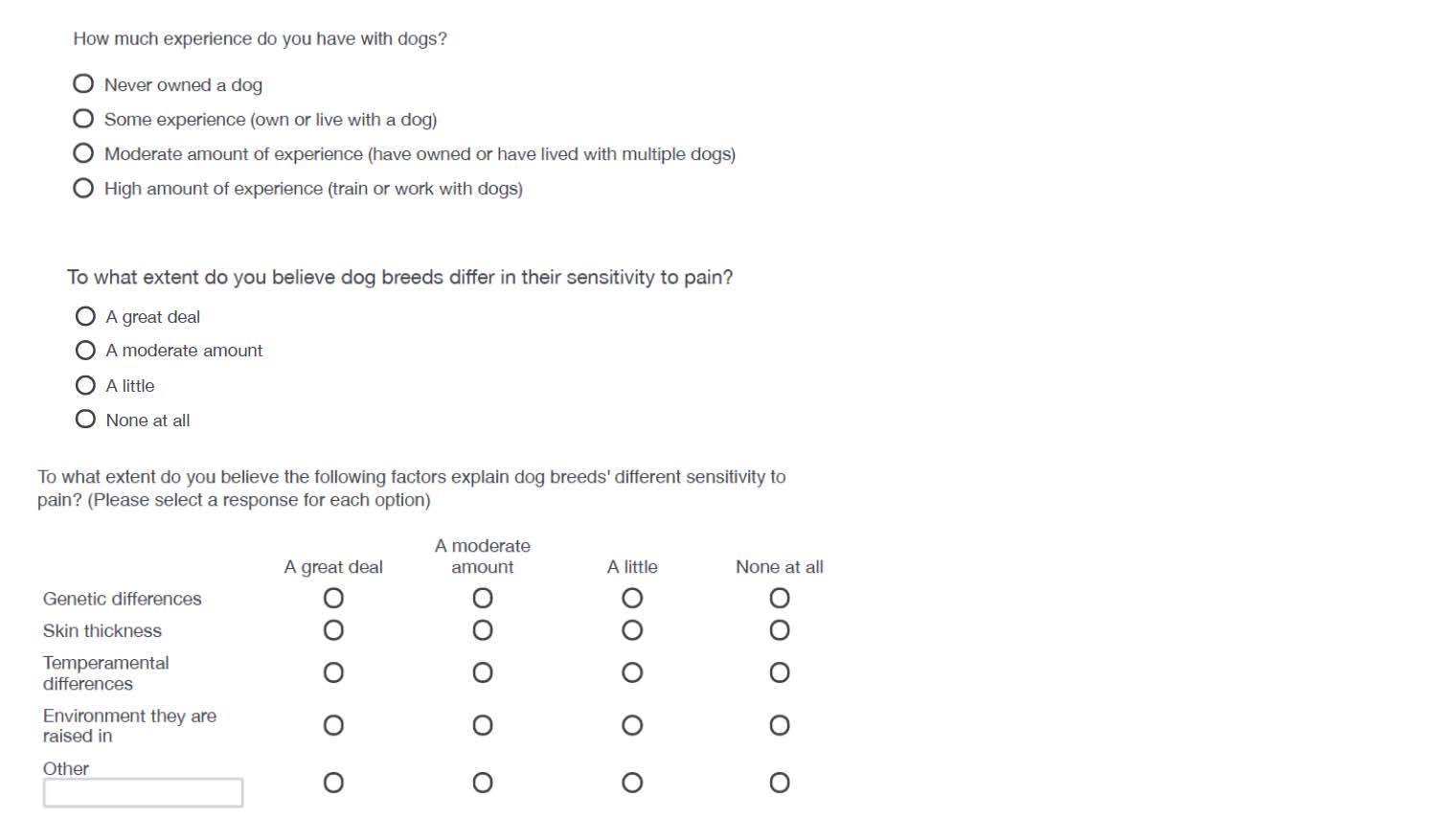


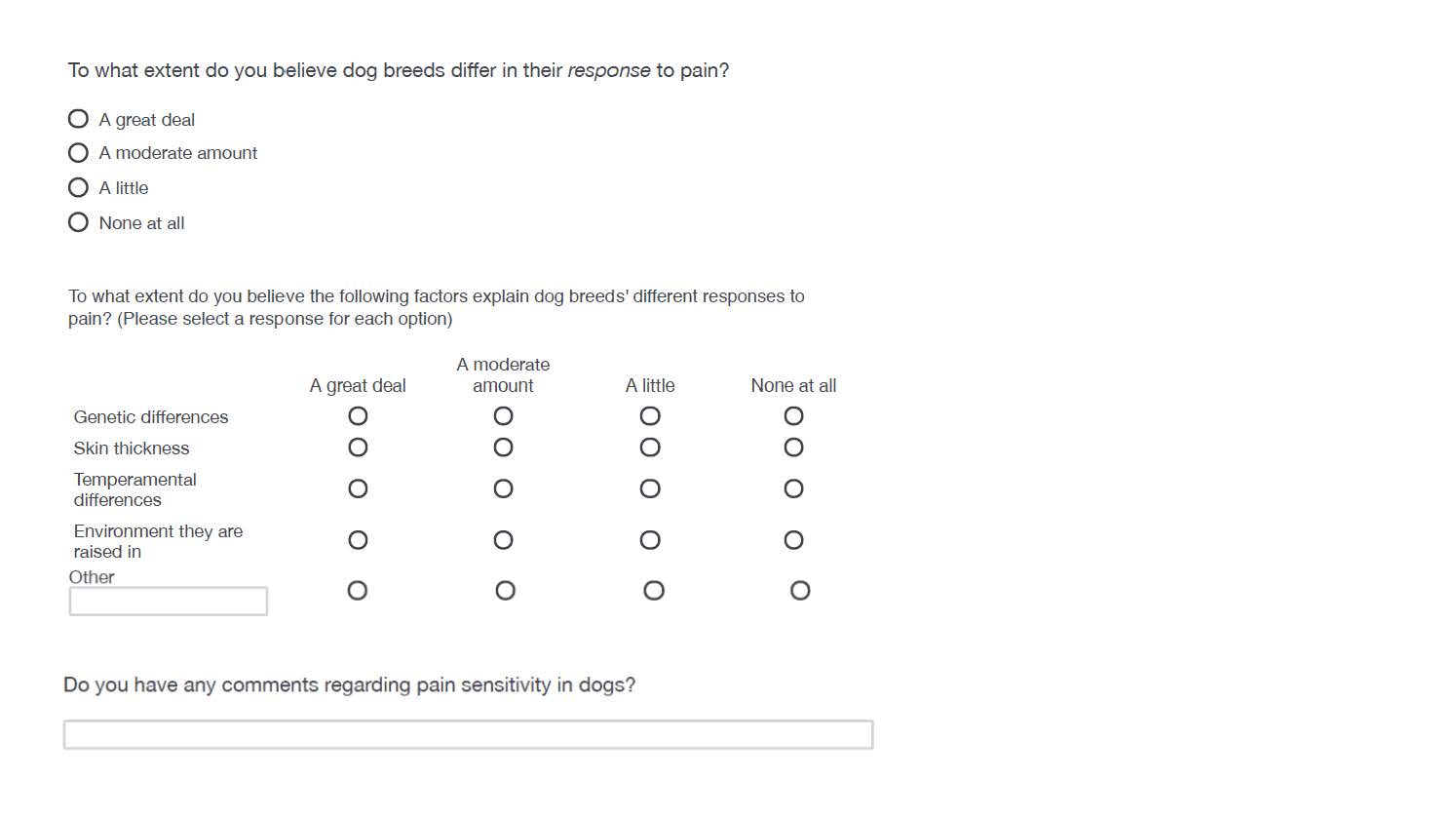


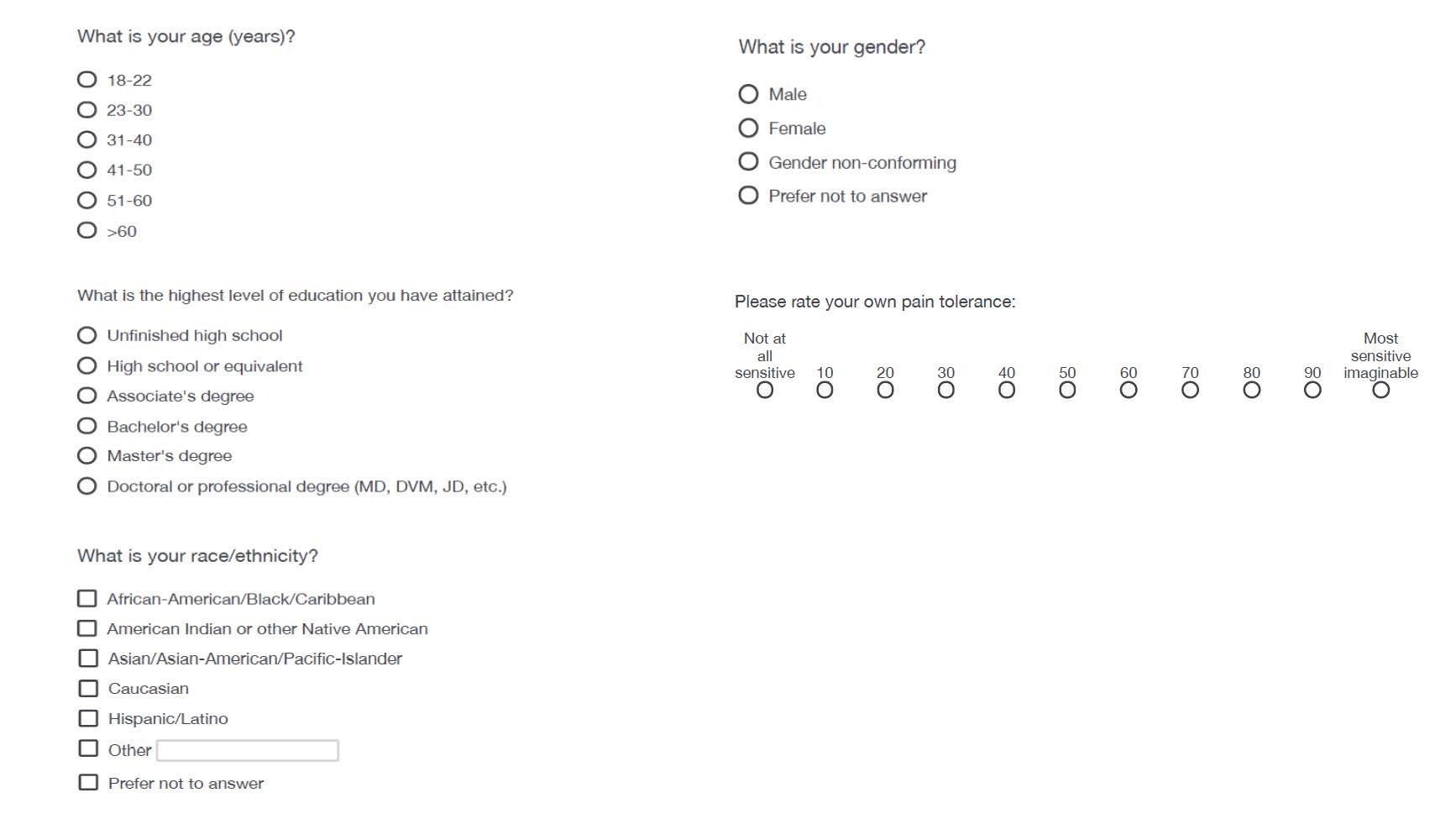


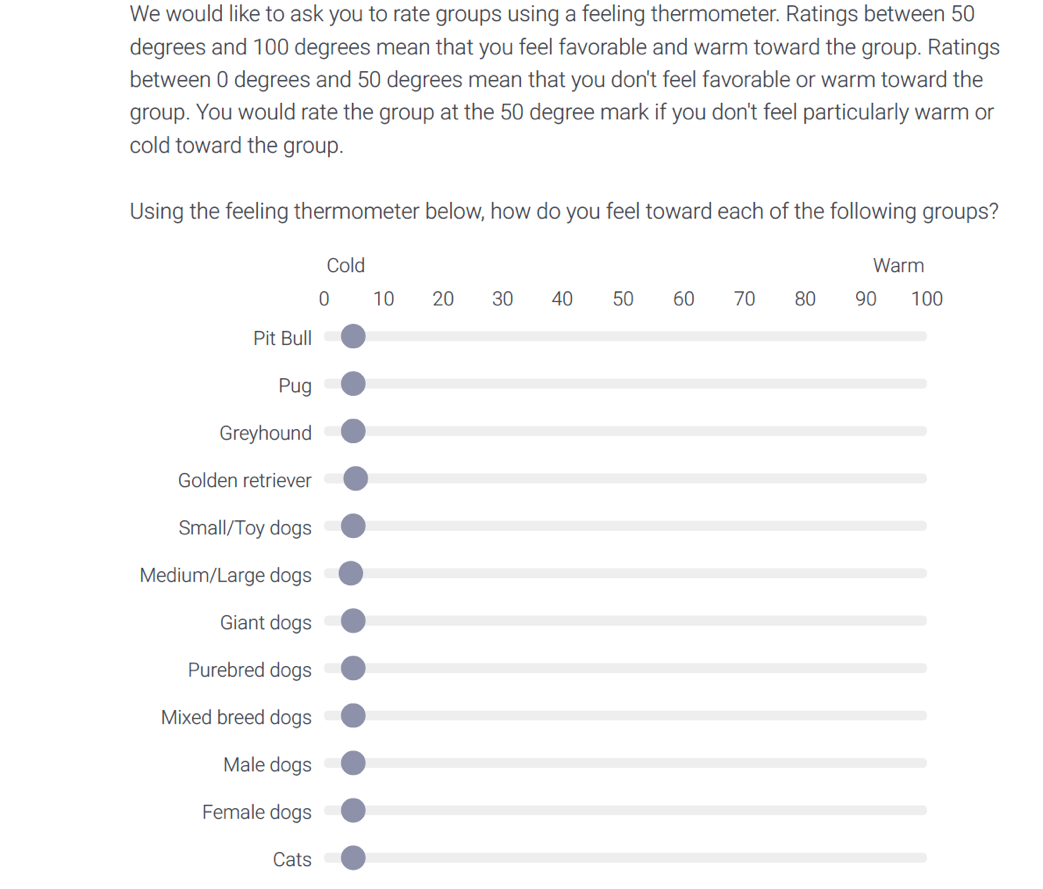

Supplement: S1 File — (DOCX) [file pone.0230315.s001.docx]
